# Camel Mastitis: Prevalence, Risk Factors, and Isolation of Major Bacterial Pathogens in Gomole District of Borena Zone, Southern Ethiopia

**DOI:** 10.1155/2021/9993571

**Published:** 2021-08-31

**Authors:** Minda Asfaw Geresu, Shubisa Abera Leliso, Galma Wako Liben

**Affiliations:** ^1^Department of Veterinary Science, College of Agriculture and Environmental Science, Arsi University, Asella, Ethiopia; ^2^National Animal Health Diagnostics and Investigation Center (NAHDIC), Sebeta, Ethiopia; ^3^Gomole District Pastoral Office, Borena Zone, Ethiopia

## Abstract

As of other dairy animals, dromedary camel could be affected by mastitis, a complex disease occurring worldwide among dairy animals, with heavy economic losses largely due to clinical and subclinical mastitis. Yet, little is known about the occurrence and potential risk factors exposing to lactating camel mastitis in Ethiopia. Consequently, a cross-sectional study was carried out from November 2018 to April 2019 so as to determine the prevalence, associated risk factors, and major bacterial pathogens causing mastitis in traditionally managed lactating camels in Gomole district of Borena Zone. Consequently, 348 lactating camels were examined for clinical and subclinical mastitis, using California Mastitis Test (CMT). The overall prevalence of mastitis was 22.4% (78/348), including clinical 4.3% (15/348) and subclinical 18.1% (63/348) cases, respectively, whereas the quarter level prevalence of mastitis was 16.6% (232/1,392). Of the total 1,392 examined teats, the right hind (RHQ) (4.3%, 60/1392) and left hind quarters (LHQ) (4.3%, 60/1392) were the most frequently infected quarter, whereas the left front quarter (LFQ) (3.9%, 55/1392) was the least infected quarter. Age, body condition score, and lactation stages were significantly associated (*p* < 0.05) with lactating camel mastitis prevalence among the putative risk factors. Among 312 quarters milk samples subjected to bacteriological examination, 69.9% (218/312) yielded mastitis causing pathogens, both Gram-positive and -negative bacterial isolates, while no growth was observed in 30.1% (94/312) of quarters sampled. Of the bacterial isolates obtained by culturing, *Streptococcus* spp. excluding *Streptococcus agalactiae (S. agalactiae)* (26.1%; 57/218) and Coagulase negative Staphylococci (22.9%, 50/218) were the dominant isolates identified, whereas *S. agalactiae* (3.2%, 7/218) was the least isolates obtained. The prevalence of camel mastitis in the study area was found to be considerably high. Hence, implementation of integrated approaches has great importance in the study setting for the prevention and control of mastitis so as to improve quality of camel milk, minimize economic loss, and prevent significant public health risks.

## 1. Introduction

Of more than 35 million camels in the world [1], Ethiopia has 4.5 million camels and 89% are one-humped (*Camelus dromedarius*) camels [2, 3]. The camel is a multipurpose animal that has outstanding performance in the arid and semiarid environments where browse and water are limited, and it makes an important contribution to human survival and utilization of these dry and arid lands [3, 4]. In Ethiopia, camels are mostly kept by pastoralists of Borana, Kereyu, Afar, Somali, Beja, and Rashaida, which cover more than 50% of the pastoralist area in the country [2, 5]. With its unique biophysiological characteristics, the dromedary has become an icon of adaptation to challenging ways of living in these arid and semiarid regions [6].

Camel plays a significant role as a source of milk, meat, and draft power in addition to being financial reserve and social security. Camel milk is a key food in arid and semiarid areas of the African and Asian countries where camel pastoralists prefer camel milk to other types of milk due to the fact that it is nutritious, is thirst quenching, is easily digestible, and can be preserved much longer [2, 4, 7]. Camel's milk is rich in protein, fat, minerals, and vitamins, especially in vitamin C. The high vitamin C content has significant importance to human diet particularly in dry areas where green vegetables and fruit are not readily available [4].

Additionally, the phosphorus contents are also higher in camel milk than that of other livestock. It is, therefore, evident that camel milk is superior to the milk of other domestic animals in many aspects [8]. Moreover, camel milk has been reported to possess medicinal value against various ailments such as dropsy, jaundice, spleen ailments, tuberculosis, asthma, anemia, piles, and food allergies [9, 10].

Though its milk has an ample of significance, like other dairy animals, dromedary camel could be affected by mastitis, a complex disease occurring worldwide among dairy animals, with heavy economic losses largely due to clinical and subclinical mastitis, the latter requiring indirect means of diagnosis [11]. Evidence indicates that subclinical mastitis causes suffering of the animal, reduces milk yield, alters milk properties, impairs preservation and processing, and is a public health concern for consumers of camel milk [12, 13].

Recently, however, occurrence of mastitis among lactating camel has been reported from different camel rearing countries like Somalia [14], Sudan [15], Kenya [16], Israel [17], and different parts of Ethiopia [4, 5, 18–24]. However, there is paucity of information on the prevalence of camel mastitis and its risk factors in Gomole district of Borena zone, Southern Ethiopia. To design appropriate control and prevention program in she-camel dairy herd, up to date information on the nature of mastitis and major bacterial pathogens associated with its occurrence need to be identified. Therefore, this study was conducted to determine the prevalence of camel mastitis to identify the major bacterial pathogens contributing to mastitis and risk factors associated with mastitis occurrence in traditionally managed lactating camels in Gomole district of Borena Zone.

## 2. Materials and Methods

### 2.1. Study Area

The study was conducted from November 2018 to April 2019 in Gomole district of Borana Zone, Oromia Regional state, Southern Ethiopia. Generally, Borena area represents a vast lowland area in Southern Ethiopia covering about 95,000 km^2^. The area is bordering with Kenya to the South, Somali region to the East, Guji zone to the North and Southern People, Nation and Nationalities Region to the West. Gomole district is located at altitude of 1857 meters above sea level, 4°52ʹN 38°5ʹE latitude, and 4.884°N 38.082°E longitudes, in the southern part of Ethiopia at about 530 km away from Addis Ababa in southern direction. Borena plateau gently slopes from high mountain massifs, 1650 masl in the North to 1000 masl in the South bordering Kenya, with slight variation due to central mountain ranges and scattered volcanic cones and craters [25].

The climate is generally semiarid with annual average rainfalls ranging from 300 mm in the south to >700 mm in the north. The rain pattern is of a bimodal type with the main rainy season called *Ganna* extending from mid-March to May and the small rainy season (*Hagayya*) from mid-September to mid-November. The other two seasons are the cool dry season (*Adolessa)* extending from June to August and the major dry season (*Bonna*) extending from December to February, whereas annual mean daily temperature varies from 19 to 24°C with moderate seasonal variation [26].

Animal husbandry in the Zone is characterized by extensive pastoral productions system and seasonal mobility. Cattle are the dominant animal species followed by goats, camels, and sheep.

According to Borana Zone Department of Planning and Economic Development Bureau, the total camel population of Borena zone was estimated to be about 450,570 of which about 30,113 camel population were found in the Gomole district [27]. Seasons affect herding strategies due to its effect on forage and water resource availability. As aridity increases, the principal stock shifts gradually from cattle combined with small stock to camels combined with small stock, with a relative degree of the social and cultural values accounting for differences. Camel herd movement may move the whole herd to water points and to relatively better areas where green fodder is available, or by herd splitting where lactating and young animals are kept around homesteads and moving the rest to distantly located forage areas [28, 29].

### 2.2. Study Population

The study animals consisted of indigenous breeds of *Camelus dromedarius* reared under pastoral management system which allows free grazing, usually mixed with livestock from other villages, the animals move from feed shortage area to feed abundant areas especially during drought season. The population consisted of lactating camels residing in Gomole district that were managed under pastoral production systems. The study animals were selected from the population at satellite livestock camps (“*Fora*”) and base livestock camps (“*Warra*”).

### 2.3. Study Design

A cross-sectional study supported by questionnaire survey was conducted to determine the prevalence of camel mastitis, to identify the major bacterial pathogens contributing to mastitis and its associated risk factors in Gomole district of Borena zone.

Questionnaire survey was conducted to assess the management aspects and possible risk factors contributing to mastitis occurrence and milk handling method with each selected lactating camel owners/herders. Data were collected by four (4) animal health extension workers through face-to-face exit interview. Structured and pretested questionnaire was used which is prepared in English and then translated to local language, “Afaan oromoo,” by the third (3^rd^) author who knows the accent of the local community. One-day training was given for the 4 animal health extension workers by emphasizing on the purpose of the study, significance, and appropriate meanings of each question, as well as the art of interviewing the participants. Individuals owned lactating camels from each “*Kebele*” (the smallest administrative unit in Ethiopia). livestock satellite and base camps were selected by the data collectors. Then, the selected lactating camel owners were interviewed, and their responses were recorded by the interviewer. The potential risk factors such as age, body condition score, lactation stage, and “*Kebeles*” (origin) were given due attention while interviewing camel owners/herders. The age of the camels was estimated using rostral dentition [30] and then categorized as young (<5 years) and adult (≥5 years of age), and body condition score of the camels was assessed according to Faye et al. [31] and then grouped as poor (score 1), medium (score 2 and 3), and good (score 4), whereas lactation stage was categorized into three categories as early (1-2 months), middle (3–9 months), and late (10–18 months) to see if there is any significant difference in the occurrence of mastitis during these stages [23] for ease of data analysis.

### 2.4. Sample Size Determination and Sampling Strategy

The number of animals sampled was calculated according to Thrusfield [32] considering a minimum expected prevalence of 50%, the desired absolute precision level of 5%, and a confidence level of 95%. A previous study conducted by Wubishet et al. [24] revealed an overall prevalence of 37.4% camel mastitis in Borena zone. Hence, the sample size for the study animals in the study area was determined using the standard formula indicated below by considering 37.4% as expected prevalence:

(1) $$\begin{array}{c} n=\frac{1.96^{2\text{Pexp}\left(1-\text{Pexp}\right)}}{d^{2}}, \end{array}$$

where *n* = the required sample size, 1.96^2^ = the value of *Z* at confidence level, Pexp = expected prevalence (50%) and *d* = the desired absolute precision level at 95% confidence interval (0.05). Accordingly, 348 lactating camels in the study site were considered in this study.

Gomole district was purposely selected for the study by considering its largest camel population, camel milk marketing, and accessibility of infrastructures. In the district, there were 14 “*Kebeles*,” of these four, namely, Dase-Gora, Buya, Bildim, and Kela-Kufa “*Kebeles*” were selected purposely by their proximity to roads, accessibility of infrastructure and camel holdings of each “*Kebele*.”

Prior to commencement of the study, lists of households (HH) of those Kebeles (sampling frame) was obtained from the district Agricultural and Rural development office, and then the HHs were randomly selected with lottery system. From each HH proportional number of lactating camels was selected by simple random sampling for collection of milk sample and physical examination. If the selected HH had no lactating camels, the next HH in the list was included till 348 camels were obtained. The sample size of HHs was determined using the formula recommended by Arsham [33] for survey studies:

(2) $$\begin{array}{c} N=\frac{0.25}{{\left(\text{SE}\right)}^{2}}, \end{array}$$

where *N* is sample size and SE is standard error of the proportion. Assuming the standard error of 7.9% at a precision level of 5%, and the confidence interval of 95%, 40 HHs owning lactating camel were selected by a simple random sampling technique for interview. The numbers of HHs selected per Kebeles were fixed based on the proportion of HHs owning lactating camel in each Kebele.

### 2.5. Clinical Examination of the Udder and Milk Sample Collection

Animals were individually identified, and clinical examination of udder was performed by visualization and palpation. During examination, palpation of udder and visual observation of udder lesion, clinical mastitis, udder symmetry, and size, as well as observation of milk consistency, color changes, and presence of grossly visible substances, were performed. Clinical mastitis was defined as an udder quarter with visible abnormal inflammatory changes in the mammary gland tissue such as redness, swelling, pain, or increased heat and/or visible inflammatory changes in the milk such as a change in color (watery, bloody, blood-tinged, serum-like, etc.) or a change in consistency (clots or flakes, or stringy or viscous) [4, 34, 35]. Moreover, blind teats were also considered as clinical cases as the blockage of the teat is the chronic stage of mastitis, which can be clinically diagnosed. Contrarily, subclinical mastitis was characterized by apparently normal milk and increased leukocyte counts. It causes cost loss because the quantity production decreases through somatic cells count (SCC) increase. The presence of SCC affects milk production reversibly, so when it increases the milk yield decrease and vice versa. To detect subclinical mastitis, milk let-down was initiated by allowing the calf to suckle for a short time, prior to milking, and then quarter milk was screened for inflammation using the California Mastitis Test (CMT) [36].

The milk samples were collected according to the sterile milk sampling protocol explained by Kirk [37]. First, sterile tube was labeled, and the udder was cleaned and dried using cotton. Then, the end of each teat was sanitized with 70% alcohol starting from the teat that is farthest away to the nearest one and 1–2 streams of milk from each teat were removed. Finally, 75% of the sterile sample tube was filled with the milk samples, which are first taken from the nearest one. It was then transported to laboratory using icebox and placed in a refrigerator at 4°C for less than 72 hours before further processing.

### 2.6. California Mastitis Test

California Mastitis Test was performed before taking milk samples for bacteriological culturing. This test was conducted after discarding the first streaks of milk; following this, about 10 mL of milk per quarter was milked into the CMT paddle, and then visual assessment of the milk was performed, with respect to consistency, color, and clots. The milk was then mixed with an equal amount of 3% CMT fluid and blended using a circular motion. Scores represented four categories: 0, negative (-) or trace (±); 1, positive (+); 2, positive (++) and 3, positive (+++). Negative (-) and trace (±) reactions were considered as “negatives” and different intensities of positive reactions (+, ++, +++) were considered as “positives” [22].

### 2.7. Bacterial Isolation and Identification

**Figure 1:**
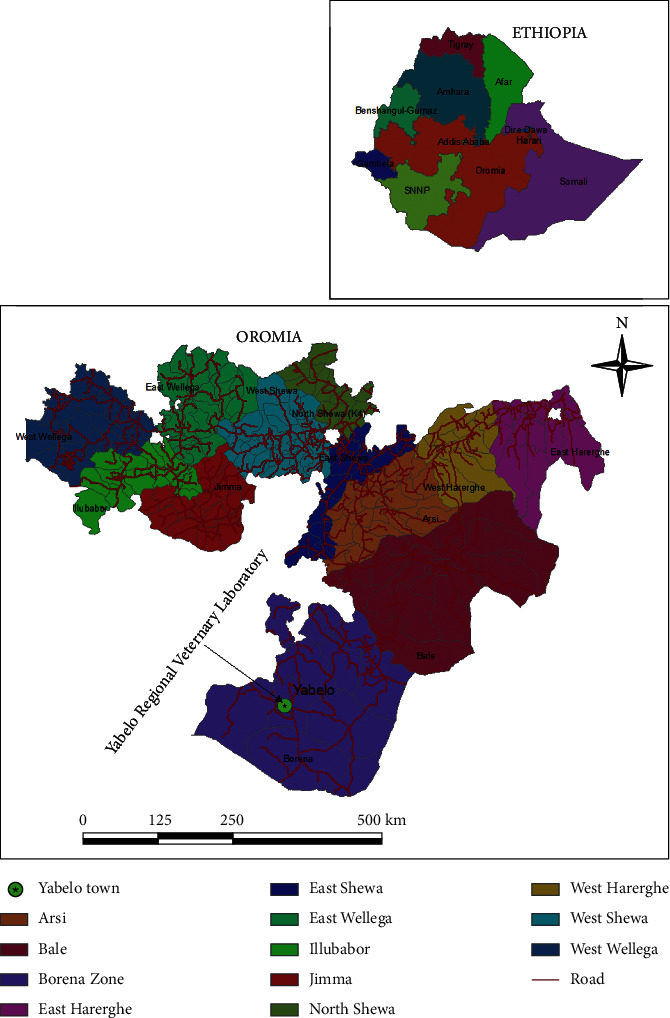
Map of Yabello regional veterinary laboratory.

The milk samples that were positive for CMT were kept in an icebox and transported immediately to Yabello Regional Veterinary Laboratory (Figure 1), and then 10 *μ*L of milk from each sample was cultured on sheep blood agar and MacConkey agar (Oxoid Ltd., Cambridge, UK) for bacteriological analysis. Inoculated plates were incubated aerobically at 37°C and evaluated for the growth of bacteria at 24 and 48 h of incubation. Then, presumptive identification of bacterial isolates was carried out based on colony morphological features, Gram-staining reactions, hemolytic reactions, catalase test, potassium hydroxide (KOH) test, and other biochemical tests [38]. Pure culture of ≥5 colony forming unit (CFU) was recorded as significant with the exception of *S. agalactiae* and *S. aureus* which were classified as significant if ≥ 1 CFU was present.

Then, bacterial isolates were transferred to their respective selective media for further characterizations and species identifications. Gram-positive cocci were tested for catalase, and catalase-positive isolates were further tested for coagulase production. Briefly, Staphylococci spp. were identified based on their growth characteristics on mannitol salt agar, coagulase, catalase, and oxidase tests. *S. aureus* was differentiated from other *Staphylococcus* species by coagulase test and maltose fermentation test. Streptococci isolates were evaluated based on CAMP reaction, hydrolysis of esculin and sodium hippurate, catalase production, and sugar fermentation tests. Specifically, *S. agalactiae* was differentiated from other mastitis-causing streptococci by using CAMP test, esculin hydrolysis on Edwards medium, and growth on MacConkey agar. Gram-negative isolates were further tested using triple sugar iron (TSI), IMViC, motility, urea, and oxidase test.

### 2.8. Data Storage and Analysis

Data generated from questionnaire survey and laboratory investigations were recorded and coded using Microsoft Excel spreadsheet (Microsoft Corporation) and analyzed using SPSS version 24.0. An overall prevalence of mastitis was calculated as the number of clinical and subclinical mastitis cases divided by the total number of samples tested. Association of prevalence with the potential risk factors (age, body condition score, origin (“*Kebele's*), and lactation stage) were computed by Chi-square (*χ*^2^) test. Then, logistic regression was conducted so as to detect the strength of association of the exposing risk factors towards the prevalence of lactating camel mastitis. Finally, associations were reported as being statistically significant whenever the *p* value was <0.05.

## 3. Results

### 3.1. Animal and Kebele Level Prevalence of Clinical and Subclinical Mastitis in Lactating Camels

Out of 348 traditionally managed lactating camels examined for mastitis, clinical as well as subclinical cases, an overall prevalence of 22.4% (Table 1) was recorded of which 4.3% and 18.1% camels were found to be affected with clinical and subclinical mastitis, respectively, as depicted in Table 2. Among the “*Kebeles*” selected from Gomole district, Buya “*Kebele*” had relatively the highest prevalence of lactating camel mastitis (11.2%), whereas Kela kufa and Bildim “*Kebeles*” (3.4%) had the lowest prevalence of clinical and subclinical mastitis amid the four selected *Kebele's* (Table 3).

### 3.2. Quarter Level Prevalence of Mastitis in Traditionally Managed Lactating Camels

Of 1392 examined quarters, 232(16.6%) quarters were found positive using CMT for subclinical mastitis and by physical examination for the clinical mastitis through excluding the blind teat from which the milk sample was not collected. The result further revealed that the right-hind (RHQ) and left-hind quarters (LHQ) were the most frequently mastitis exposed quarters (4.3%), whereas the left-front quarter (LFQ) was the least exposed quarter (3.9%) as indicated in Table 4.

### 3.3. Putative Risk Factors Associated with the Occurrence of Mastitis in Lactating Camels

A Chi-square analysis revealed that age, body condition score, and lactation stages were significantly associated (*p* < 0.05) with lactating camel mastitis prevalence among the putative risk factors considered during the study as depicted in Table 5.

### 3.4. Household's Questionnaire Survey Result

Locally, udder health problem is known as “*dhukkuba muchaa*,” which literally means ‘disease of teats. Though the name implies “disease of teat,” the term is understood to be general udder health problems. Pastoralists associated the problems of udder health with different factors and grouped based on the perceived causes and clinical signs into different categories. The main categories identified were “*diraandisa*” (tick infestation), “*nyaqarsa*” (chronic swelling in the form of a boil), and “*Buda*” (which means evil eye and is characterized by bloody milk).

Of the 40 HHs owning camels interviewed, 85% (34/40) of them responded that as clinical mastitis is the major problem and a disease, they were aware of while all of them were not aware of subclinical mastitis. All of the HHs interviewed responded that as milk ejection was initiated by letting the calves to suckle their dams before milking, washing the udder/teats of camels is not practiced prior to milking, milking utensils were washed and smoked before milking and treat lactating camel mastitis cases by a combination of phytotherapeutics and modern antimicrobials. Particularly, experienced (elder) camel owners indicated that they know traditional ways of treating camel mastitis using traditional folk remedies. Of the respondents, 95% (38/40) were using local herbal medicine known as “*Aloe vera*” to treat the disease by topical application on swollen udder. Pertaining to season of the occurrence of the disease, the majority of the respondents (85%, 34/40) stated that the disease was mostly occurring during wet major rainy season (“*Ganna*”) and early lactation stage, while only 12.5% (5/40) of the respondents were responding as it occurs during short rainy season (“*Hagayya*”), whereas none of the respondents indicated that if the disease was occurring during cold dry season (“*Adolessa*”) and the major dry season (“*Bona*”).

### 3.5. Bacterial Isolation and Identification

Among 312 quarter milk samples subjected to bacteriological examination, 218 (69.9%) yielded mastitis pathogens, both Gram-positive and -negative bacteria isolates, while no growth was observed in 94 (30.1%) quarter samples. Of the bacterial isolates, *Streptococcus* spp. excluding *S. agalactiae* (26.1%) and Coagulase negative Staphylococci (22.9%) were the dominant isolates identified, whereas *S. agalactiae* (3.2%) was the least as illustrated in Table 6.

## 4. Discussion

Mastitis is an important constraint to milk production in pastoralist camel (*Camelus dromedarius*) herds in arid and semiarid parts of Ethiopia and a number of reports revealed that mastitis in traditionally managed camels is increasing and likely continues to rise as the milk production per individual camel gradually increases [39]. Accordingly, the overall prevalence of mastitis in camel herds (animal level) in the current study, 22.4%, is lower than the prevalence report of 59.8% in Afar Region of Ethiopia [19], 76% in selected pastoral areas of eastern Ethiopia [22], 18.5% in Abu Dhabi, United Arab Emirates [40], 30.2% in Jijiga town of eastern Ethiopia [5], 44.8% in Yabello district of Borena Zone [23], 34.7% in Borena zone of Oromia Regional State [24], and 31% in Gursum district of Hararghe Zone [40]. Besides this, the prevalence of mastitis at quarter level observed in this study is also lower than the reports of Husein et al. [5], Almaw and Molla [18], and Zeryehun et al. [41], whom reported 25.8%, 20.5%, and 25.6% at the quarter level using CMT, respectively. Nevertheless, Abera et al. [4] reported a relatively lower (15.8%) of subclinical mastitis from Eastern Ethiopia compared to the present study.

On top of this, the current study results also revealed that the hind quarters, right hind (RH, 4.3%) and left hind (LH, 4.3%), were inflamed more compared to the front quarters, the right (RF, 41%) and left front (LF, 3.9%), which contradicts the finding of Mogeh et al. [42] who recorded that the right quarters, right (RF, 15.6% and RH, 7.8%), were highly exposed compared to the left quarters (LF, 5.2% and LH, 4.6%). A lower prevalence in the current study could be due to variation in the sensitivity of diagnostic CMT screening techniques used, season of study, and absence of bush clearing in the study area, which prevents the grass to grow that hosts more ticks, whereas a higher risk of infections in hind quarters compared to the front ones could be due to the unfavorable hygienic condition, greater exposure to dung and urine. In addition, due to the shorter length of the hind teats with a corresponding shorter teat canal, the defense potential in the hind quarter could be decreased [43].

Thus, the finding of clinical mastitis (4.3%) in the current study is higher than the prevalence report of 2.1% from Borena lowland pastoral area, Southwestern Ethiopia [20]. Conversely, the current finding is relatively lower than the report of 4.9% in Jijiga town of eastern Ethiopia [5], 5.4% in Borena zone of Oromia Regional State [23], 5.7% in pastoral area of Borena lowland [41], 6.3% in Gursum district of eastern Hararghe zone [40], 8.3% in Jijiga, Eastern Ethiopia [4], 12.5% in Afar region [19], 12.5% in Borena Zone [24], and 19.5% in Eastern Sudan [44]. Pertaining to the subclinical mastitis, a prevalence rate of 18.1% at animal level, the present work result is relatively higher than the report of Osman [44] who reported a prevalence rate of 15.8% from Jijiga zone of Somali Regional State of Ethiopia compared to the present study. Nevertheless, the current study result is lower than reports of Abera et al. [4], Husein et al. [5], Almaw and Molla [18], Bekele and Molla [19], Regassa et al. [23], Wubishet et al. [24], and Mehamud et al. [40] whom reported 20.7%, 25.3%, 24.1%, 47.3%, 25.4%, 22.2%, and 24.7% at camel levels using CMT from Jijiga Zone of Somali Regional State, around Jijiga in eastern part of Ethiopia, eastern Ethiopia, Afar region, Borena zone, and Gursum district of eastern Hararghe zone, respectively. This difference might be due to the season of the study period (dry season) and absence of tick infestation load related with season of study.

The current study revealed that the selected *Kebeles'* from Gomole district of Borena Zone was negatively associated with lactating camel mastitis (*p* > 0.05). Of the 4 selected *Kebeles*, the highest prevalence of lactating camel mastitis was observed in Buya *Kebele* (11.2%), whereas Kela kufa and Bildim *Kebeles* (3.4%) had the lowest prevalence of lactating camel mastitis among the selected study sites. Hence, this area needs an indebt study to unveil the factors responsible for this difference.

Age was considerably associated with the prevalence of mastitis as detected by CMT and microbiological culturing which corroborates with the finding of Zeryehun et al. [41] and Aqip et al. [45] who reported that adult age (≥5) was found significantly (*p* < 0.05) associated with the occurrence of lactating camel mastitis in pastoral area of Borena lowland of South-western Ethiopia and in Cholistan desert of Pakistan, respectively.

The nonparametric statistical analysis revealed that medium body condition score of the animals was positively associated (*p* < 0.05) with the occurrence of lactating camel mastitis in the current study in which there was no mastitis case in poor (thin) body condition score. This finding is inconsistent with the report of Zeryehun et al. [41], Aqib et al. [45], and Ali et al. [46] whom reported a significant association of thin body condition score with the occurrence of mastitis in dromedary camel in Cholistan desert of Pakistan, pastoral area of Borena lowland of Southern Ethiopia, and Cholistan desert and Suleiman Mountain range of Pakistan, respectively. Hence, this area needs an indebt study to unveil the factors responsible for this difference.

Likewise, stage of lactation significantly affected (*p* < 0.05) and was found to be associated with the prevalence of mastitis being the highest (52.9%; 54/102) during the early stage of lactation. This finding corroborates with the findings of Husein et al. [5], Ahmad et al. [9], Regassa et al. [23], and Mogeh et al. [42] whom revealed a positive association of early lactation stage with the incidence of camel mastitis in desert environment of Jhang (Pakistan), Jijiga town, eastern part of Ethiopia, Borena zone and Hargeisa district, western part of Somaliland, respectively.

About 85% (34/40) of interviewed respondents (pastoralists) stated that camel clinical mastitis is the major problem and the disease they are aware of. This finding relatively corroborates with the finding of Husein et al. [5] who stated that about 70% of the respondents were aware of clinical mastitis and as they know it by different names in Jijiga, eastern Ethiopia, whereas all of them were not aware of subclinical mastitis which agrees with the reports of Abera et al. [4] and Husein et al. [5] from Jijiga town, eastern Ethiopia. On top of this, all the households interviewed in the study setting responded that as milk ejection was initiated by letting the calves to suckle their dams before milking which agrees with the work of Seligsohn et al. [47] who stated that calves were released one by one and allowed to suckle their mothers to initiate milk let-down. Moreover, as all the interviewed respondents responded that milk ejection was initiated by letting the calves to suckle their dams before milking and milking utensils were washed and smoked before milking camels which is consistent with the reports of Husein et al. [5] and Seifu and Tafesse [22] in selected pastoral areas of eastern Ethiopia.

Out of 40 HHs owning camels interviewed, 85% (34/40) of them responded that as clinical mastitis is the major problem and a disease they were aware of, while all of them were not aware of subclinical mastitis. All of the HHs interviewed in the study setting responded that as milk ejection was initiated by letting the calves to suckle their dams before milking, washing the udder/teats of camels is not practiced prior to milking and milking utensils were washed and smoked before milking camels, whereas almost all (95%) of the respondents interviewed stated that as they were using local herbal medicine plant known as “*Aloe vera*,” applied topically on the swollen udder, and modern antimicrobials to treat the diseases of udder in the present study. Our finding corroborates with the report of Abera et al. [4] who reported as clinical mastitis was treated by a combination of phytotherapeutics and modern drugs in Jijiga town. In contrast to this, Seifu and Tafesse [22] reported that camel owners were using various extracts from the roots, leaves, seeds, and exudates of different plant and branding with hot iron in selected pastoral areas of eastern Ethiopia. Pertaining to season of the occurrence of the disease, the majority of respondents (85%) stated that the disease was mostly occurring during wet major rainy season (“*Ganna*”) and early lactation stage while only 12.5% of the respondents responded that it occurs during short rainy season (“*Hagayya*”), whereas none of the respondents indicated whether the disease was occurring during cold dry season (“*Adolessa*”) and the major dry season (“*Bona*”).

The commonly isolated genera of bacteria *Staphylococcus*, *Streptococcus*, *Corynebacterium*, *Bacillus*, and *Escherichia* in this study agree with [4, 19, 39, 48–51] whom isolated *Staphylococcus*, *Streptococcus*, and *Escherichia* as major mastitogens. The isolation rate of *E. coli* in the current study relatively verifies the finding of Mengistu et al. [52] and Alebie et al. [53]. As coliforms can be a sign of inadequate hygienic conditions and to a minor degree of fecal contamination [54], the prevalence may vary considerably according to hygiene conditions.

The occurrence of *S*. *aureus* (11.92%) in this study is much higher than the finding of Almaw and Molla [18] who reported 0.6% but lower than the reports of Woubit et al. [20] and Mengistu et al. [52] who reported 21.03% and 16%, respectively. Such variation might attribute to traditional taboo on heat treatment of camel milk and maintaining milk at high ambient temperature after milking and during transportation in the study area can pose a serious problem to human health as these practices create conducive situation for the production of staphylococcal enterotoxin Alebie et al. [53], whereas, of the total isolates, 22.94% of coagulase-negative Staphylococci (CNS) detected in CMT positive milk samples closely agrees with the findings of Woubit et al. [20] (18.2%) and Alebie et al. [53] (19.57%). Nevertheless, it is lower than Mengistu et al. [52] who reported 40.4%. Though it is reported that these Staphylococci spp. are known as facultative (“minor”) pathogens isolated from subclinical mastitis cases which do not show a measurable influence on milk yield, CMT, or clinical symptoms [54], an explanation for their frequent occurrence is most probably due to the contamination of the milk samples by the teat canal or teat skin.

The highest occurrence of *Streptococcus* spp. excluding *S. agalactiae* in this study is much higher than the report of Alamin et al. [15] and Hadef et al. [55] whom reported a prevalence of 1.52% and 2.38% from North Kordofan State of Sudan and Southeastern Algeria, respectively. However, it is much lower than previous studies conducted by Saleh and Faye [51] in Al-Jouf, Saudi Arabia (42.9%), whereas the lower prevalence (3.21%) of *S. agalactiae* reported in the current study substantiates with the report of Husein et al. [5] who reported a prevalence of 3.5% from Jijiga town of Ethiopia but lower than the report of Seligsohn et al. [47], Mehamud et al. [40], and Al-Tofaily and Al Rodhan [56] whom reported a prevalence of 72%,10% and 9.52% from Isiolo of Kenya, Gursum district of eastern Hararghe, Ethiopia, and some areas of middle Euphrates in Iraq, respectively. The low proportion of *S. agalactiae* might be attributed to medication of the animal's mastitis cases by a combination of traditional folk remedies and modern antimicrobials in the study setting.

The occurrence of *Bacillus* spp. in the current report (10.5%) is higher than the report of Mehamud et al. [40] and Mengistu et al. [52] whom reported 6.6% and 4.3% of the cases from Gursum district of eastern Hararghe and Gewane district of Afar Regional State, Ethiopia, correspondingly, but lower than the report of Alebie et al. [53] who reported a higher prevalence of 19.57% from Dubti district of Afar Regional State, North-eastern Ethiopia. *Micrococcus* spp. isolates (7.34%) recovered from this is closely in line with the report of Alebie et al. [53], Saleh and Faye [51], Mengistu et al. [52], and Woubit et al. [20] whom reported a prevalence of 4.35%, 5.7%, 6.4%, and 10.58% from Dubti district, Afar Regional State of Ethiopia, Al-Jouf, Saudi Arabia, Gewane district, Afar Regional State of Ethiopia and pastoral area of Borena, southwestern Ethiopia, congruently. Furthermore, the prevalence of *Corynebacterium* spp. (9.2%) in the current investigation corroborates with the report of Husein et al. [5] who reported a prevalence of 9% from Jijiga town of Ethiopia. Nonetheless, this finding is higher than the report of Alamin et al. [15] from North Kordofan State of Sudan ((3.03%), whereas the occurrence of *Trueperella pyogenes (T. pyogenes)* (5.05%) in this study contradicts the report of Seligsohn et al. [47]. The occurrence of different bacterial species reported in the current study could be due to poor milking hygiene (washing the udder/teats of camels is not practiced prior to milking) in the study area.

## 5. Conclusion

The current study result revealed that the prevalence of camel mastitis in the study area was found to be considerably high. The study revealed that a relatively higher teat quarter subclinical and clinical mastitis, of which the right and left hind quarters were the most frequently acquiring mastitis. Age, body condition score, and lactation stages were significantly associated with lactating camel mastitis prevalence among the putative risk factors considered in the study. *Streptococcus* spp. (24.6%) and Coagulase negative *Staphylococci* (21.6%) were among the dominant major bacterial isolates identified, whereas *Streptococcus agalactiae* was the least isolates obtained in this study. The bacteria isolated from camel milk samples in the present study are types that cause both contagious and environmental mastitis. Proper and worthy milking techniques are essential in the prevention of both environmental and contagious mastitis. Therefore, in order to reduce a relatively high prevalence of mastitis in the area, improved milking hygiene, washing of udder/teat, and treating of clinically infected she-camels with the available folk medicine and modern antimicrobials should be practiced.

## Figures and Tables

**Table 1:** Prevalence of mastitis at the animal and quarter level based on the CMT in Gomole district of Borena zone.

Status	Number of examined animals	Number of positives	Prevalence (%)
Camel level	348	78	22.4
Quarter level	1392	232	16.6

**Table 2:** Prevalence of clinical and subclinical form of mastitis at animal level in Gomole district of Borena zone.

Form of mastitis	Number of positives	Prevalence (%)
Clinical	15	4.3
Subclinical	63	18.1
Total	78	22.4

**Table 3:** Prevalence of clinical and subclinical mastitis at *Kebele* level in the selected study district.

*Kebele's*	Number of animals examined	Number of positives	Prevalence (%)
Dase-Gora	56	15	4.3
Buya	187	39	11.2
Kela-Kufa	57	12	3.4
Bildim	48	12	3.4
Total	348	78	22.4

**Table 4:** Quarter level prevalence of mastitis in traditionally managed lactating camels in the study site.

Quarter	Number of blind teats	Number of positives	Prevalence (%)
Right front	15	57	4.1
Right hind	8	60	4.3
Left front	7	55	3.9
Left hind	11	60	4.3
Total	41	232	16.6

**Table 5:** Prevalence of mastitis in association with different putative risk factors in lactating camels in the study site.

Risk factors		Number of camels examined	Number of positives	*p* value
*Kebeles*	Dase-Gora	56	15	0.772
	Buya	187	39	
	Kela-Kufa	57	12	
	Bildim	48	12	
Age	Young (<5 years)	248	69	0.000
	Adult (≥5 years)	100	9	
Body condition score	Good	138	33	0.001
	Medium	168	45	
	Poor	42	0	
Lactation stage	Early	102	54	0.000
	Mid	98	3	
	Late	148	21	

**Table 6:** Bacterial species isolated from quarter milk samples obtained from traditionally managed lactating camels in the selected study area.

Bacterial species	Number of isolates	% of total isolates
*Escherichia coli*	23	10.5
*S. aureus*	26	11.9
*S. agalactiae*	7	3.2
*Bacillus* spp.	8	3.7
Coagulase negative Staphylococci	50	22.9
*Streptococcus* spp. excluding *S. agalactiae*	57	26.1
*Micrococcus* spp.	16	7.3
Corynebacteria spp.	20	9.2
*Trueperella pyogenes*	11	5.1
Total	218	100

## Data Availability

The data that support the findings of this study are available from the corresponding author upon reasonable request.

## References

[1] Faye B. (2020). How many large camelids in the world? A synthetic analysis of the world camel demographic changes. *Pastoralism*.

[2] Abera T., Legesse Y., Mummed B., Urga B. (2016). Bacteriological quality of raw camel milk along the market value chain in Fafen zone, Ethiopian Somali regional state. *BMC Research Notes*.

[3] Mohammed Y. K., Seid A., Urge M. (2017). Camel (*Camelus dromedaries*) meat production potentials and associated constraints in Eastern Ethiopia. *East African Journal of Veterinary and Animal Science*.

[4] Abera M., Abdi O., Abunna F., Megersa B. (2010). Udder health problems and major bacterial causes of camel mastitis in Jijiga, Eastern Ethiopia: implication for impacting food security. *Tropical Animal Health and Production*.

[5] Husein A., Haftu B., Hunde A., Tesfaye A. (2013). Prevalence of camel (*Camelus dromedaries*) mastitis in Jigjiga town, Ethiopia. *African Journal of Agricultural Research*.

[6] El-Agamy E. I., Park Y., Haenlein G. F. W. (2006). Camel milk. *Handbook of Milk of Non-bovine Mammals*.

[7] Mohammed A. H. (1993). Conceptual classification of camels. *The Multipurpose Camel: Interdisciplinary Study on Pastoral Production in Somalia*.

[8] Kouniba A., Berrada M., Zahar M., Bengoumi M. (2005). Composition and heat stability of Moroccan camel milk. *Journal of Camel Practice and Research*.

[9] Ahmad S., Yaqoob M., Bilal M. Q. (2011). Risk factors associated with prevalence and major bacterial causes of mastitis in dromedary camels (*Camelus dromedarius*) under different production systems. *Tropical Animal Health and Production*.

[10] Shabo Y., Barzel R., Margoulis M., Yagil R (2005). Camel milk for food allergies in children. *The Israel Medical Association Journal: The Israel Medical Association Journal*.

[11] Matofari J. W., Mario Y., Mwatha E. W., Okemo P. O. (2003). Microorganisms associated with subclinical mastitis in Kenyan camels (*Camelus dromedarius*). *Journal of Tropical Microbiology and Biotechnology*.

[12] Tibary A., Anouassi A., Skidmore L., Adams G. P. (2000). Reproductive disorders in the female camelids. *Recent Advances in Camelid Reproduction*.

[13] Gramay S., Ftiwi M. (2018). Camel milk production, prevalence and associated risk factors of camel mastitis in Asaita Woreda, Afar Regional State, North East Ethiopia. *ARC Journal of Animal and Veterinary Sciences*.

[14] Mohamud A. I., Mohamed Y. A., Jama O. S. A., Mishra P., Mohamed M. I. (2020). Prevalence and major pathogens associated with clinical and subclinical mastitis in dairy camel (*Camelus dromedarius*) in Benadir Region of Somalia. *Veterinary Sciences: Research Review*.

[15] Alamin M. A., Alqurashi A. M., Elsheikh A. S., Yasin T. E. (2013). Mastitis incidence and bacterial causative agents isolated from lactating she-camel (*Camelus dromedaries*). *IOSR Journal of Agriculture and Veterinary Science*.

[16] Kashongwe O. B., Bebe B. O., Matofari J. W., Huelsebusch C. G. (2017). Associations between milking practices, somatic cell counts and milk postharvest losses in smallholder dairy and pastoral camel herds in Kenya. *International Journal of Veterinary Science and Medicine*.

[17] Guliye A. Y., Van Creveld C., Yagil R. (2002). Detection of sub-clinical mastitis in dromedary camels using somatic cell count and the N-acetyl beta-D-Glucosominidase test. *Tropical Animal Health and Production*.

[18] Almaw G., Molla B. (2000). Prevalence and etiology of mastitis in camels (*Camelus dromedaries*) in eastern Ethiopia. *Journal of Camel Practice and Research*.

[19] Bekele T., Molla B. (2001). Mastitis in lactating camels (*Camelus dromedarius*) in Afar Region, north-eastern Ethiopia. *Berliner und Münchener Tierärztliche Wochenschrift*.

[20] Woubit S., Bayleyegn M., Bonnet P., Jean-Baptiste S. (2001). Mammites du dromadaire (Camelus dromedarius) dans la région pastorale basse du Borana au sud-ouest de l’Ethiopie. *Revue d’élevage et de médecine vétérinaire des pays tropicaux*.

[21] Abdul-Gadir A. E., Hildebrand G., Kleer J. N., Molla B., Kyule M. N., Baumann M. P. (2006). Comparison of California mastitis test, somatic cell and bacteriological examinations for detection of camel (*Camelus dromedaries*), mastitis in Ethiopia. *Berlin Munch Tierarzil Woshenschr*.

[22] Seifu E., Tafesse B. (2010). Prevalence and etiology of mastitis in traditionally managed camels (*Camelus dromedarius*) in selected pastoral areas in eastern Ethiopia. *Ethiopian Veterinary Journal*.

[23] Regassa A., Golicha G., Tesfaye D., Abunna F., Megersa B. (2013). Prevalence, risk factors, and major bacterial causes of camel mastitis in Borana Zone, Oromia Regional State, Ethiopia. *Tropical Animal Health and Production*.

[24] Wubishet Z., Dabaso A., Getachew G. (2016). Prevalence, associated risk factors and bacterial pathogens of camel mastitis in Borena Zone Oromia Regional State, Ethiopia. *International Journal of Veterinary Science*.

[25] Coppock D. (1994). *The Borana Plateau of Southern Ethiopia Synthesis of Pastoral Research, Development and Change*.

[26] Megersa B. (2010). An epidemiological study of major camel diseases in the Borana lowland, Southern Ethiopia. *The Drylands Coordination Group (DCG)*.

[27] Jara R., Alemayehu M., Wubishet Z., Mesfin T., Araya M. (2020). Sero-prevalence and associated risk factors of camel brucellosis in Southern lowland of Ethiopia. *Journal of Veterinary Medicine and Research*.

[28] Demeke G. (1998). Prevalence of camel trypanosomes and factors associated with the disease occurrence in Liben district, Borana zone of Oromia region, Ethiopia.

[29] Wario S., Wubishet Z., Alemayehu M. (2020). Prevalence and associated risk factors of major prevalent gastrointestinal nematodes in camels of Borena Zone, Southern Ethiopia. *Journal of Veterinary Medicine and Research*.

[30] Bello A., Sonfada M. L., Umar A. A. (2013). Age estimation of camel in Nigeria using rostral dentition. *Scientific Journal of Animal Science*.

[31] Faye B., Bengoumi M., Cleradin A., Tabarani A., Chilliard Y. (2017). Body condition score in dromedary camel: a tool for management of reproduction. *Emirates Journal of Food and Agriculture*.

[32] Thrusfield M. (2008). *Sampling in Veterinary Epidemiology*.

[33] Arsham H. (2007). *Questionnaire Design and Survey Sampling*.

[34] Radostits O., Gay C., Hinchcliff K., Constable P. (2007). *Veterinary Medicine: A Text Book of Disease of Cattle, Horses, Sheep, Pigs and Goats*.

[35] Balemi A., Gumi B., Amenu K. (2021). Prevalence of mastitis and antibiotic resistance of bacterial isolates from CMT positive milk samples obtained from dairy cows, camels, and goats in two pastoral districts in Southern Ethiopia. *Animals*.

[36] Schalm O. W., Noorlander D. O. (1957). Experiments and observations leading to development of the California mastitis test. *Journal of the American Veterinary Medical Association*.

[37] Kirk J. (2000). Sterile milk sampling: extension UDVM.

[38] National Mastitis Council (NMC) (1990). *Microbiological Procedures for the Diagnosis of Bovine Udder Infection*.

[39] Al-Juboori A. A., Kamat N. K., Sindhu J. I. (2013). Prevalence of some mastitis causes in dromedary camels in Abu Dhabi, United Arab Emirates. *Iraqi Journal of Veterinary Sciences*.

[40] Mehamud J., Megersa M., Abebe Y., Ahmed M. (2017). Prevalence, risk factors and major bacterial causes of camel mastitis in Gursum district, Eastern Hararghe, Ethiopia. *Global Veterinaria*.

[41] Zeryehun T., Haro G., Adane B. (2017). A cross sectional study on the prevalence of mastitis and associated bacterial pathogens in one-humped camels (*Camelus dromedarius*) in pastoral area of Borena lowland, Southern Ethiopia. *Global Veterinaria*.

[42] Mogeh A. O., Teklu A., Ogleh M. D. (2019). The prevalence of mastitis and its associated risk factors in lactating dromedary camels in and around Hargesa, Somaliland. *International Journal of Scienctific and Engineering Research*.

[43] Wanjohi M., Gitao C. G., Bebora L. (2013). Subclinical mastitis affecting hygienic quality of marketed camel milk from North Eastern Province, Kenya. *Microbiology Research International*.

[44] Osman A. (2008). Prevalence of camel mastitis and major bacterial causes in Jigjiga zone, Somalia region.

[45] Aqib A. I., Ijaz M., Durrani A. Z. (2017). Prevalence and antibiogram of *Staphylococcus aureus*, a camel mastitogen from Pakistan. *Pakistan Journal of Zoology*.

[46] Ali M., Avais M., Ijaz M. (2019). Epidemiology of subclinical mastitis in Dromedary camels (*Camelus dromedarius*) of two distinct agro-ecological zones of Pakistan. *Pakistan Journal of Zoology*.

[47] Seligsohn D., Nyman A.-K., Younan M. (2020). Subclinical mastitis in pastoralist dairy camel herds in Isiolo, Kenya: prevalence, risk factors, and antimicrobial susceptibility. *Journal of Dairy Science*.

[48] Kalla D. J. U., Butswat I. S. R., Mbap S. T., Abdussamad A. M., Ahmed M. S., Okonkwo I. (2008). Microbiological examination of camel (*Camelus dromedarius*) milk and sensitivity of milk microflora to commonly available antibiotics in Kano, Nigeria. *Savannah Journal of Agriculture*.

[49] Matofari J. W., Younan M., Nanua J. N., Mwatha E. W. (2005). Microorganisms associated with sub-clinical mastitis and their impact on milk production in camels (*Camelus dromedarius*) in semi-arid lands of Northern Kenya. *International Journal of Agriculture and Rural Development*.

[50] Sena D. S., Mal G., Kumar R., Sahani M. S. (2001). A preliminary study of prevalence of mastitis in camel. *Journal of Applied Animal Research*.

[51] Saleh S. K., Faye B. (2011). Detection of subclinical mastitis in dromedary camels (*Camelus dromedaries*) using somatic cell counts, California mastitis test and udder pathogen. *Emiratus Journal of Food and Agriculture*.

[52] Mengistu F., Molla B., Ali A. (2010). Camel mastitis, associated bacterial pathogens and its impact on milk quality in Gewane district, Afar Regional State, Northeastern Ethiopia. *Animal Health and Production*.

[53] Alebie A., Molla A., Adugna W., Tesfaye A., Ejo M. (2021). Prevalence, isolation, identification, and risk factors of major bacterial cause of camel subclinical mastitis. *BioMed Research International*.

[54] Eberlein V. (2007). *Hygienic Status of Camel Milk in Dubai (United Arab Emirates) under Two Different Milking Management Systems*.

[55] Hadef L., Aggad H., Hamad B. (2018). Bacterial causative agents associated with subclinical mastitic in dromedary she-camels in Southeastern Algeria. *Jordan Journal of Biological Sciences*.

[56] Al-Tofaily Y. I. K., Al rodhan M. A. N. (2011). Study on clinical mastitis (bacteriological) in she-camels (*Camelus dromedarius*) in some areas of middle Euphrates in Iraq. *AL-Qadisiya Journal of Veterinary Medicine Science*.

